# Serial Non-Invasive Assessment of Antibody Induced Nephritis in Mice Using Positron Emission Tomography

**DOI:** 10.1371/journal.pone.0057418

**Published:** 2013-02-27

**Authors:** Guiyang Hao, Yong Du, Xin J. Zhou, Jianfei Guo, Xiankai Sun, Chandra Mohan, Orhan K. Öz

**Affiliations:** 1 Department of Radiology, University of Texas Southwestern Medical Center, Dallas, Texas, United States of America; 2 Internal Medicine, University of Texas Southwestern Medical Center, Dallas, Texas, United States of America; 3 Department of Pathology, University of Texas Southwestern Medical Center, Dallas, Texas, United States of America; 4 Advanced Imaging Research Center, University of Texas Southwestern Medical Center, Dallas, Texas, United States of America; UC Irvine Medical Center, United States of America

## Abstract

Mouse models of experimental anti-glomerular basement membrane (anti-GBM) nephritis provide an analytical tool for studying spontaneous lupus nephritis. The potential of Positron Emission Tomography (PET) was evaluated using 2-deoxy-2-[^18^F]fluoro-d-glucose (FDG) as a probe to monitor the progression of anti-GBM induced nephritis in a mouse model. The imaging results were compared to conventional measures of renal function and pathological changes. Serum and urinary vascular cell adhesion molecule-1 (VCAM-1) levels were used as measures of endothelial cell activation and inflammation. Following a challenge with anti-glomerular antibodies, mice exhibited peak changes in serum creatinine, proteinuria, and glomerulonephritis score at 14 days post-challenge (p.c.). In contrast, VCAM levels peaked at day 7 p.c. On dynamic PET images (0–60 min) of day 7, kidneys of the anti-GBM nephritis mice demonstrated a unique pattern of FDG uptake. Compared to the time activity curve (TAC) prior to challenge, a rightward shift was observed after the challenge. By day 10 p.c., kidney FDG uptake was lower than baseline and remained so until the study ended at 21 days p.c. During this time frame measures of renal dysfunction remained high but VCAM-1 levels declined. These changes were accompanied by an increase in kidney volume as measured by Computed Tomography (CT) and intra-abdominal fluid collection. Our results suggest that FDG-PET-CT can be used as a non-invasive imaging tool to longitudinally monitor the progression of renal disease activity in antibody mediated nephritis and the magnitude of renal FDG retention correlates better with early markers of renal inflammation than renal dysfunction.

## Introduction

Systemic lupus erythematosus (SLE) is a chronic inflammatory and autoimmune disease. The Lupus Foundation of America estimates that 1.5 million Americans have lupus and at least 5 million worldwide. The average annual direct health care cost per patient with SLE was $12,643 in the USA as reported in 2008, which imposes a considerable financial burden on the nation and the patient’s family [1]. SLE can affect almost all parts of the body. Among them, renal involvement (lupus nephritis) is the foremost cause of morbidity and mortality in SLE patients [2]. Lupus nephritis is characterized by repeated episodes of flares. To date, renal biopsy remains the gold standard to diagnose and assess the disease status of lupus nephritis patients. However, due to inherent limitations of potential sampling errors and its invasive nature, multiple biopsies that are necessary for the assessment of the disease or treatment efficacy are undesirable and not routinely clinically performed. Moreover, clinically silent chronic changes of glomerulosclerosis and interstitial fibrosis secondary to chronic inflammation may go undetected with biopsy. These changes predispose to chronic kidney disease and end-stage renal disease. Therefore, it would be of clinical value to develop a non-invasive method to detect or assess renal disease.

Several animal models have been used to uncover the underlying mechanisms of human lupus nephritis [2]. Indeed, several inbred or hybrid mouse strains develop spontaneous lupus reproducibly. However, the long duration of disease development (usually 6–12 months) hampers their use in the research of the disease [3]. A more rapid model entails subjecting mice to anti-glomerular basement membrane antibody (anti-GBM) to induce experimental nephritis [2]. Although the initial insults and clinical presentation may differ in the two diseases, it has been shown that the anti-GBM nephritis model shares common downstream molecular mechanisms with spontaneous lupus nephritis [3], [4]. Moreover, the anti-GBM model can be reproducibly induced in mice within a time-frame of 2–3 weeks. This short time-frame makes it an appealing model to evaluate experimental therapies and imaging techniques.

The most commonly used PET probe, 2-deoxy-2-[^18^F]fluoro-D-glucose (FDG), is a D-glucose analog, in which the hydroxyl group at the 2′ position is replaced by ^18^F, a positron-emitting radioisotope of fluorine. After intracellular uptake, FDG is phosphorylated to FDG-6-phosphate by hexokinase. Being highly negatively charged, FDG-6-phosphate is trapped inside the cells. Because of the 2′ position substitution, this metabolite cannot be metabolized further in the glycolytic pathway or for glycogen synthesis. Therefore, FDG can be used as a surrogate to track glucose distribution and phosphorylation *in vivo* by means of PET. In addition to its success in oncology, FDG-PET has also shown promise in clinical evaluation of infection and inflammation because of the elevated glucose consumption in activated inflammatory cells [5]–[7]. For example, FDG-PET could provide high sensitivity (77–92%) and specificity (89–100%) predicative information for the diagnosis of large-vessel vasculitis in untreated patients with elevated inflammatory markers [8]. Unlike D-glucose, following glomerular filtration, deoxyglucose and FDG are incompletely reabsorbed by the renal tubules after intravenous administration. The unresorbed FDG appears in the renal collecting system and urine [7]. Therefore, dynamic imaging of the kidney permits identification of abnormal kinetics within the renal cortex or the collecting system. We hypothesized that experimental lupus nephritis might alter FDG uptake and/or clearance kinetics.

In this study, we evaluated the potential of FDG-PET as a non-invasive imaging technique to longitudinally monitor the renal disease status in an anti-GBM nephritis mouse model.

## Materials and Methods

All animal studies were reviewed and approved by the Institutional Animal Care and Use Committee at the University of Texas Southwestern Medical Center, Dallas, Texas.

### Mice and Anti-GBM Nephritis Model

The mice (129×1/SvJ) were purchased from the Jackson Laboratory. All mice were maintained in a specific pathogen–free colony. Two to 3-month-old female mice were used for all studies. To induce anti-GBM nephritis, we first sensitized the mice on day 0 with rabbit IgG (250 µg/mouse, intraperitoneal injection), in adjuvant, as described previously [9]. On day 5, the mice were challenged intravenously with rabbit anti-GBM IgG (200 µg per 25 g body weight, in a 300 µL volume).

### Determination of Renal Function and Pathological Changes

Blood and 24 h urine samples were collected on days 0, 7, 10, 14, and 21 for the measurement of serum creatinine (sCr) and proteinuria, respectively. Three to five animals were sacrificed on day 0, 7, 14, and 21, respectively. The kidneys were excised and processed for histopathological examination by light microscopy. Three micrometer sections of formalin-fixed, paraffin-embedded kidney tissues were cut and stained with periodic acid-Schiff (PAS). The evidence of pathological changes in the glomeruli, tubules, or interstitial areas was examined in a blinded fashion. The glomeruli were screened for evidence of hypertrophy, proliferative changes, crescent formation, hyaline deposits, fibrosis/sclerosis, and basement membrane thickening. Likewise, the tubulointerstitial injury was gauged by the presence of tubular atrophy, inflammatory infiltrates, and interstitial fibrosis.

### ELISA Detection of Serum and Urine VCAM-1

Serum and urine VCAM-1 level was measured by ELISA. The kits were purchased from R&D Systems (Minneapolis, MN) and were used according to the manufacturer’s instructions. All serum samples were diluted 1∶100 and urine sample 1∶5.

### Mouse PET-CT Imaging

Small animal PET-CT imaging studies were performed on days 0, 7, 10, 14, and 21 on a Siemens Inveon PET-CT Multimodality System. All mice were fasted of food overnight before scan. Ten minutes prior to imaging, the animal was anesthetized using 3% isofluorane at room temperature until stable vital signs were established. Once the animal was sedated, it was placed onto the imaging bed under 2% isofluorane anesthesia for the duration of imaging. The CT imaging was acquired at 80 kV and 500 µA with a focal spot of 58 µm. After the CT scan, the mouse was injected intravenously with ∼ 37 MBq (100 µCi) of FDG and a 0–60 min dynamic PET was immediately performed. Reconstructed CT and PET images were fused and analyzed using the manufacturer’s software. For PET quantification, the regions of interest (ROI) were selected to include the whole right and left kidneys guided by the CT images. The resulting quantitative data were expressed as %ID/g. The calculation of AUC in the FDG renograph was performed by integrating the area in the time frame 0–30 min.

### RT-PCR Analysis

Quantitative polymerase chain reaction (qPCR) was performed to detect the mRNA expression of SGLTs in the kidney tissue. After isolating total RNA using TRIzol, first-strand reverse transcription reactions were performed on 1 µg of total RNA using the TaqMan reverse transcription reagent (Applied Biosystems, Foster City, CA). mRNA levels were determined by real-time PCR using the Fast SYBR® Green Master Mix (Applied Biosystems) on a Bio-Rad CFX96™ real-time system (Bio-Rad, Hercules, CA). Expression of all genes was normalized to cyclophilin A-2 expression using the standard ΔC_T_ method. The primer sequences used for amplification of SGLT1, 2, 3a, and 3b are listed in Table 1.

**Table 1:** The primer sequences used for amplification of SGLT1, SGLT2, SGLT3a, and SGLT3b.

		Primer sequence
**SGLT1**	Forward	5′- TGGAGTCTACGCAACAGCAAGGAA -3′
	Reverse	5′- AGCCCACAGAACAGGTCATATGCT-3′
**SGLT2**	Forward	5′- AGAATGGAGCAACACGTAGAGGCA -3′
	Reverse	5′- ACCAGCAGGAAATAGGCAGCGATA -3′
**SGLT3a**	Forward	5'- TGTTGGTTGGGTCCTTCATCCTCA -3'
	Reverse	5'- ACCTGGAGTTGATGGTCAGGTTGT -3'
**SGLT3b**	Forward	5'- ACATTGGCAGCAATCACTTCGTGG -3'
	Reverse	5'- AAACACCCAACCAAGAACCAGCAC -3'
**Cyclophilin A2**	Forward	5'- GCGTTTTGGGTCCAGGAATGGCA -3'
	Reverse	5'- GAGCAGATGGGGTAGGGACGC-3'

### Statistical Analysis

Statistical analyses were performed by unpaired two-tailed *t* test using GraphPad Prism 5.0. Differences were considered to be statistically significant at *p*<0.05. All results are presented as mean ± standard deviation.

## Results

### Renal Function and Pathological Changes in Anti-GBM Challenged 129×1/SvJ mice: Inflammatory Changes and Progressive Renal Failure

**Figure 1:**
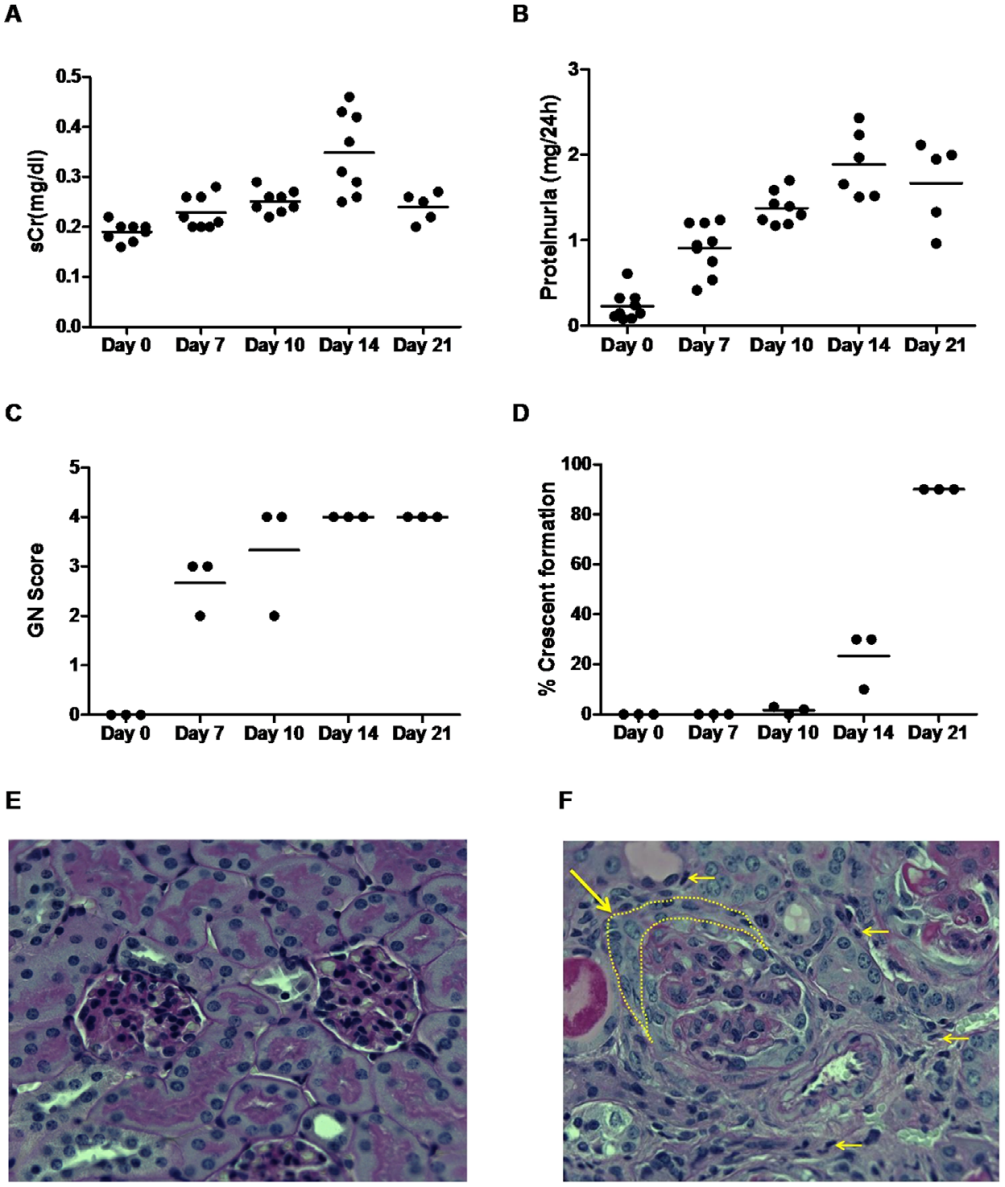
Renal dysfunction and pathological changes during anti-GBM antibody–induced nephritis. Following the challenge with anti-GBM serum, 129×1/SvJ mice (*n* = 3–9 per group) showed increased serum creatinine (sCr) levels (A) and proteinuria (B); Kidney specimens from all anti-GBM nephritis mice were examined by light microscopy for evidence of glomerular nephritis (GN score) (C), and glomerular crescent formation (D). Representative Periodic Acid Schiff (PAS) staining on day 0 (E) and day 14 (F) (yellow dash line indicates the formation of crescent). F also shows severe inflammatory cell infiltration in the tubular-interstitial area of anti-GBM nephritic mice as indicated by yellow arrows. The severity of GN score was graded on a 0–4 scale as follows: 0, normal; 1, mild increase in mesangial cellularity and matrix; 2, moderate increase in mesangial cellularity and matrix, with thickening of the GBM; 3, focal endocapillary hypercellularity with obliteration of capillary lumina and a substantial increase in the thickness and irregularity of the GBM; and 4, diffuse endocapillary hypercellularity, segmental necrosis, crescents, and hyalinized end-stage glomeruli.

The anti-GBM nephritis mouse model was successfully established in 129×1/SvJ female mice as verified by renal function analysis and pathology. Both serum creatinine (sCr) and proteinuria peaked on day 14, and subsided thereafter (Figure 1A and 1B). In order to clarify the pathological changes in the diseased animals, three to five mice in the anti-GBM nephritis group were sacrificed on days 0, 7, 10, 14, and 21. Concurrent with the renal dysfunction, the anti-GBM nephritis mice exhibited increased glomerulonephritis (GN) (Figure 1C), dramatic crescent formation (Figure 1D) as well as tubulointerstital lymphatic infiltration (Figure 1E–F) after the anti-GBM serum challenge as compared to the mice prior to anti-GBM nephritis inducement. Starting from day 14, obvious fluid accumulation in the abdominal cavity was observed during the dissection.

### PET-CT Imaging and Quantitative Analysis: Altered Renal t_max_, Area Under the Nephrogram Curve, and Appearance of the Steady State on Day 7 in Anti-GBM Mice

**Figure 2:**
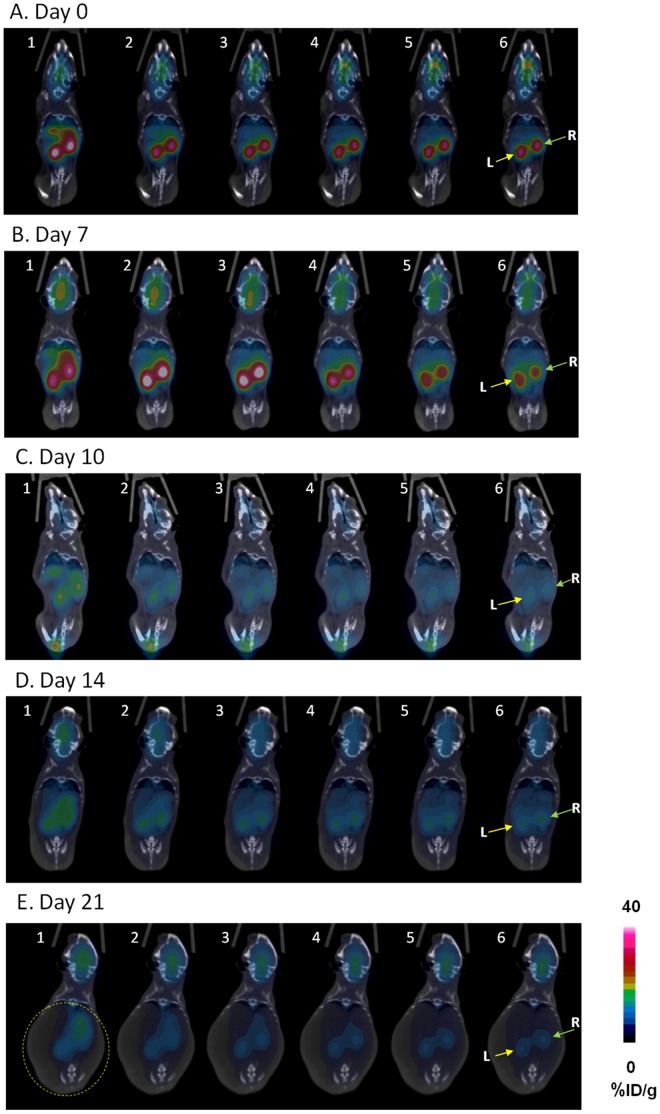
Representative coronal PET-CT images of mice following FDG injection. Images were obtained 0 (A), 7 (B), 10 (C), 14 (D) and 21 days (E) post-administration of rabbit IgG injection. They were derived from the 0–60 min dynamic scans (5 min per frame and 12 frames in total). L: left kidney; R: right kidney. The yellow dashed circle delineates the substantial enlargement of the abdominal cavity on day 21.

**Figure 3:**
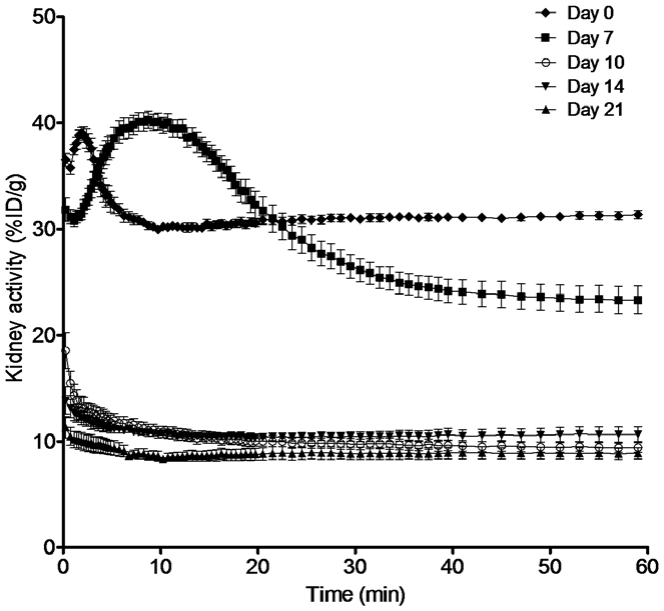
Quantitative analysis of PET-CT images. Time-activity curves of FDG in kidneys (left and right kidneys pooled) reconstructed at short time intervals (0–1 min: 30 s; 1–5 min: 15 s; 5–20 min: 30 s; 20–40 min: 60 s; 40–60 min: 120 s). Day 0, 7, 14, and 21: *n* = 4–5. Error bars are the standard deviation. The defined nephritis characteristic phase is visualized in 0–30 min.

In order to evaluate the potential of FDG-PET to monitor the progression of renal disease activity in the anti-GBM nephritis mouse model, we performed a longitudinal PET-CT imaging study. On day 0, the mice were scanned prior to the rabbit IgG injection. On each study day, each mouse underwent a whole-body dynamic FDG-PET-CT scan immediately after the intravenous injection of FDG to 60 min post injection (p.i.) (*n* = 4–5). To better illustrate overall FDG uptake and distribution changes during the dynamic imaging, panels of coronal PET-CT images captured at 5 min intervals are presented in Figure 2A–E. Because major changes in renal activity were observed from 0 to 30 minutes, only the first six time-frame images are shown for each day of imaging. On day 0, the kidney uptake of FDG quickly reached the maximum level within the first 5 min p.i., followed by rapid clearance, and attainment of plateau/steady state (Figure 2A). On day 7, images in mice challenged with the rabbit anti-GBM IgG showed prolonged renal retention of FDG, with higher intensity of activity than in the mice on day 0 at frames 2–4 (Figure 2A–B), consistent with a time activity shift. Renal FDG uptake substantially decreased on day 10 and 14 (Figure 2C–D, Figure 3). With worsening renal function, abdominal swelling became obvious upon physical examination, which was also clearly demonstrated on the PET-CT images from the nephritic mice (Figure 2E).

Since the left and right kidneys showed nearly identical FDG uptake, their uptake values were pooled for quantitative data analysis. Shown in Figure 3 are the time-activity curves (TAC) of FDG signal captured over the whole kidney through imaging analysis at short time intervals (0–1 min: 30 s; 1–5 min: 15 s; 5–20 min: 30 s; 20–40 min: 60 s; 40–60 min: 120 s). Consistent with the visual observations, the mice on day 0 exhibited the highest renal FDG activity measured by percent injected dose per gram of tissue (%ID/g) at 1.9±0.5 min (t_max_) followed by a rapid decline and then a slower prolonged plateau/equilibrium phase. Compared to untreated mice of day 0, the antibody treated mice demonstrated a unique pattern of renal TACs on day 7, consisting of a rightward shift in the time to peak and a prolonged second phase with slower decline in renal activity, shown summarily as longer intra-renal retention time. Additionally, the plateau or steady state phase was of higher amplitude on day 7 (Figure 3). The kidney t_max_ appeared at 8.7±3.8 min on day 7 for the anti-GBM mice. On days 10, 14, and 21, the t_max_ decreased to the time immediately after injection but most impressively the amplitude of maximum renal uptake values were significantly lower (*p*<0.0001). For further analysis, we quantified the area under the TACs from 0 to 30 min. The area under the curve (AUC) during this nephritis-characteristic phase increased from 948±14%ID·min·g^-1^ on day 0 to 1022±31%ID·min·g^-1^ on day 7, and then decreased to 327±18%ID·min·g^-1^ on day 10, 325±12%ID·min·g^-1^ on day 14, and 270±17%ID·min·g^-1^ on day 21 (Table 2).

**Table 2:** PET imaging parameters and renal function/pathological changes in anti-GBM nephritis mice.

	Parameter	Day 0	Day 7	Day 10	Day 14	Day 21
***PET imaging analysis***						
	Uptake_max_ (%ID/g)	39.0±0.5	40.3±0.8	18.5±1.7*	13.8±1.3*	11.3±1.0*
	t_max_ (min)	1.9±0.5	8.7±3.8	<1.0	<1.0	<1.0
	AUC (%ID·min·g^−1^)	948±14*	1022±31	327±18*	325±12*	270±17*
***Renal function/pathological changes***						
	sCr (mg/dl)	0.190±0.019*	0.229±0.033	0.251±0.230	0.349±0.082*	0.240±0.029
	Proteinuria	0.229±0.171*	0.909±0.295	1.376±0.190*	1.886±0.389*	1.672±0.500*
	GN score	0	2.7±0.6	3.3±1.1	4.0±0*	4.0±0*
	% Crescent formation	0	0	2.0±0.7*	23±12*	90±0*
	VCAM-1 (serum)	305172±46956*	736638±136727	439871±64455*	321336±57250*	474569±108318*
	VCAM-1/Creatinine(urine)	23±22*	513±229	793±164	880±353	283±304

Uptake_max_: the maximum kidney uptake; t_max_: the corresponding time of Uptake_max_; AUC: the area under the time-activity curve during the disease characteristic uptake phase (0–30 min). sCr: serum creatinine; BUN: blood urea nitrogen; GN score: glomerulonephritis score. Data was shown as mean±standard deviation.

Note: The symbols indicate significant differences compared to *Day 7* data under the same parameter with *p<0.05.

**Figure 4:**
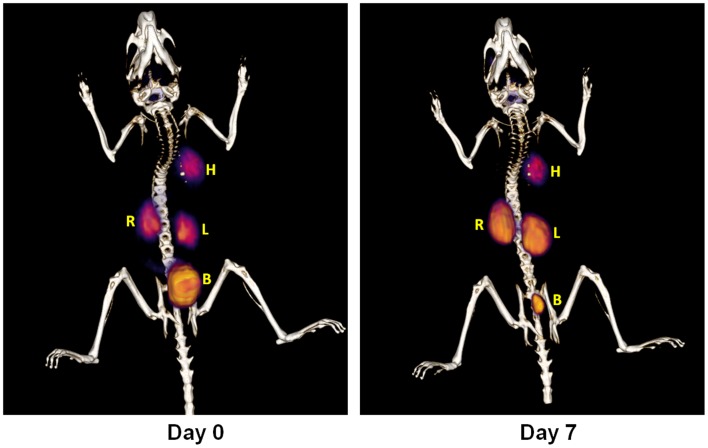
Representative 3D PET-CT images from the dynamic imaging interval of 10–15 min (frame No.3) on days 0 and 7 in anti-GBM nephritis group mice. Left: Day 0 (prior to rabbit IgG injection); Right: Day 7. H - heart, L - left kidney, R - right kidney, B - bladder.

The difference in kidney uptake at day 0 and day 7 could also be appreciated on 3D PET-CT images (Figure 4). While the mice showed much higher PET signal intensity in kidneys on day 7 than on day 0 at frame 3 (10–15 min), similar uptake levels were observed in the heart on day 0 and day 7, with negligible uptake elsewhere. Concurrently, the mice had much lower urine activity in the bladder on day 7 than on day 0. At the later time points the renal dysfunction was accompanied by low-level diffuse activity in the abdominal cavity (data not shown).

### Renal Weight and Volume Increases in Anti-GBM Nephritis Mice

**Figure 5:**
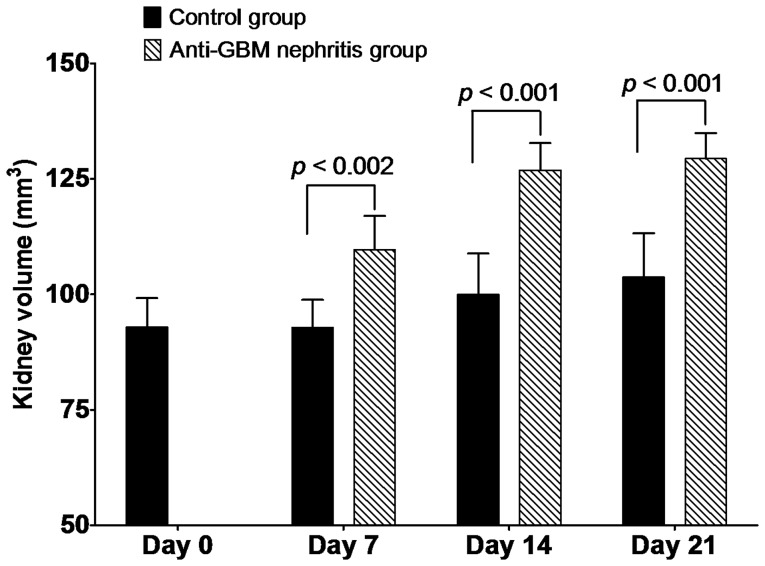
CT image derived kidney volume. Renal volume was calculated from PET-CT scans using manufacturer’s software (*n* = 3 per group, per time point). The rate of rise in kidney volume in the nephritis group clearly exceeded the increase that might be associated with growth in the control group.

It is known that inflammation results in edema. Therefore we measured the wet weights of the kidneys. The kidney weight was 0.32±0.03 g (body weight: 21.8±0.4 g) in the diseased mice as compared to 0.26±0.01 g (body weight: 18.5±2.0 g) in the controls harvested on day 21. Prompted by the *ex vivo* observation, we quantified the kidney size of both groups of mice by the 3D reconstructed CT images. To demonstrate an advantage of non-invasive imaging to show changes in renal size, we also measured the kidney volume on 3D CT images in a separate study, in which one group of mice (*n* = 3) were induced with anti-GBM nephritis and the other (*n* = 3) were injected with PBS as control. As shown in Figure 5, while both groups of mice showed an increase of their kidney size over the period of study, the anti-GBM nephritis mice had significantly enlarged kidneys compared to the corresponding control mice.

### Increased Serum and Urine VCAM-1 in Anti-GBM Nephritis Mice

Vascular cell adhesion molecule 1 (VCAM-1) is an endothelial adhesion and inflammatory molecule that has been reported to play an important role in lupus nephritis [10], [11]. Indeed, the urinary VCAM-1 level has been shown to be a good marker of renal disease in both anti-GBM disease and spontaneous lupus nephritis [12]. Hence, we examined the relationship between the serum and urine VCAM-1 levels and the FDG uptake following anti-GBM disease. As summarized in Table 2, following anti-GBM disease induction, serum VCAM-1 peaked on day 7 and then gradually declined thereafter. Likewise, urinary VCAM-1 rapidly increased >20-fold within the first seven days and continued to rise thereafter. Since renal FDG retention peaked at day 7, the peak FDG correlates with peak serum VCAM-1 levels, a marker of endothelial cell activation and inflammation.

### Alterations of Glucose Transporters in Anti-GBM Nephritis Mice

**Figure 6:**
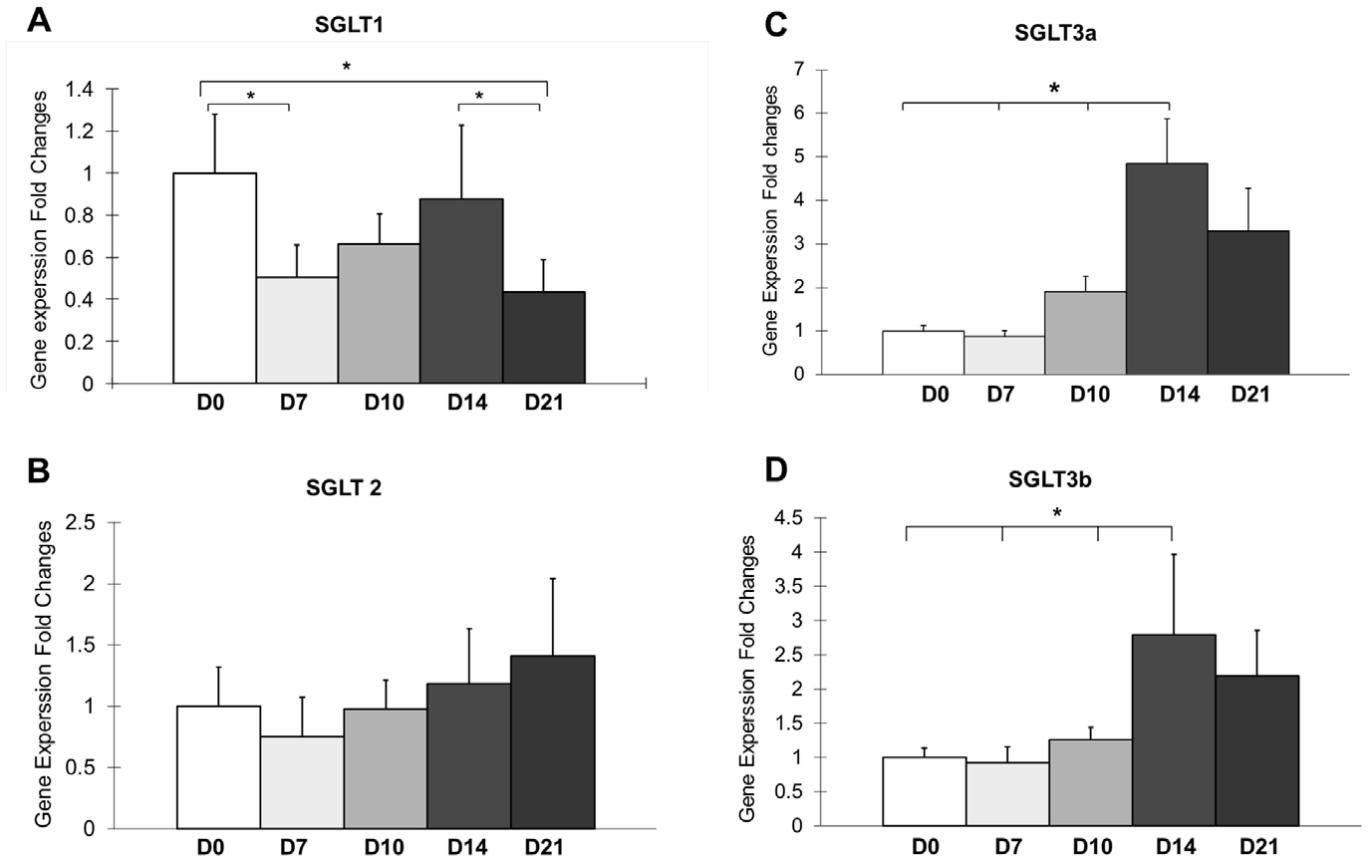
Quantitative RT-PCR analysis of SGLT1 (A), SGLT2 (B), SGLT3a (C), and SGLT3b (D) expression in the kidneys on day 0, 7, 10, 14, and 21. The values on day 0 are normalized to 1. Fold change is relative to day 0. *p<0.05, n = 3.

Recently, it was reported on studies of an *in vitro* model that in the course of kidney reabsorption the transport of FDG was predominantly mediated by sodium glucose co-transporters (SGLTs), while the transport of D-glucose is mediated by both sodium-independent glucose transport proteins (GLUTs) and SGLTs [13]. Therefore, we analyzed the temporal expression of the known SGLT1, 2, 3a, and 3b. Relative to day 0 expression levels, there was a significant decline in SGLT1 expression in kidney 7 days after anti-GBM treatment (Figure 6A). SGLT2 also declined but the drop was not significant (Figure 6B). On the other hand, there was progressively increased expression of all SGLTs on days 10 and 14 (Figure 6A–D). With the exception of SGLT2 the increase began to reverse on day 21.

## Discussion

Currently, the gold standard for diagnosis and the treatment follow up of patients with lupus nephritis is histologic evaluation of invasive biopsies, which often results in patient morbidity, especially when multiple biopsies are performed. Therefore, there is an unmet clinical need for non-invasive imaging techniques that would enable longitudinal assessment of disease progression, evaluation of acute flares, and response to treatment in the same subject. Ideally the imaging study would take advantage of current understanding of the underlying pathophysiology. Lupus nephritis is initiated by the glomerular deposition of autoantibodies (e.g. anti-GBM antibodies) and immune complexes. This triggers a cascade of inflammatory events including upregulation of adhesion molecules on endothelial cells (including VCAM-1), activation of intrinsic renal cells, recruitment of inflammatory cells, release of various inflammatory mediators, and eventual fibrosis [2], [14]. Our model recapitulated many of these findings. Recruited inflammatory cells were observed within the kidneys of the anti-GBM nephritis mice, VCAM-1 expression was elevated and renal failure ultimately developed.

We selected two parameters, t_max_ and AUC, that could be conveniently derived from the quantitative analysis of clinically acquired PET images to evaluate the nephrographs as a potential indication of the status of anti-GBM induced nephritis. The trends of t_max_ and AUC changes within the 21-day study period were parallel to the changes in renal function and pathological changes in the anti-GBM nephritic mice, but most importantly the FDG uptake peak preceded the clinical disease peak by ∼1 week. As shown in Figure 1, the clinical phenotype (proteinuria, serum creatinine) and pathological phenotype (GN score) peaked on day 14 and subsequently exhibited a self-limiting course as reported [15], [16]. In comparison, the FDG renal retention peaked on day 7 (Table 2) where both t_max_ and AUC were greatest over our study period. Of particular interest, the occurrence of these changes in renal FDG retention coincided with systemic endothelial cell activation and inflammation and the early phase of renal inflammation, as marked by the rapid increase in serum and urine VCAM-1 by day 7 (Table 2). To our knowledge this is the first study showing a correlation between VCAM-1 expression and FDG uptake/retention in the kidneys.

The mechanism of FDG handling in the kidney is complex and is not fully understood [13], [17]. Like glucose, FDG accumulates in the kidney and then undergoes glomerular filtration. Unlike glucose, the two-stage reabsorption of FDG in the renal tubular proximal segment is incomplete with some excreted through the urine [18]. Nephrographs or TACs reflect the perfusion of the kidney at the earliest time points followed by impact of glomerular filtration, tubular reabsorption and secretion, and finally clearance from the renal pelvis. As is evident from the nephrograph on day 0, FDG accumulated then rapidly cleared through kidneys followed by a prolonged plateau phase. In contrast, on day 7 the anti-GBM nephritic mice showed a delayed uptake with peak shift, increased t_max_ (8.7 min on day 7 vs. 1.9 min on day 0), prolonged tubular concentration phase, less rapid excretion rate leading to delayed onset of the plateau phase. This contributes to the increased AUC. In our model, many factors could have contributed to the alteration of FDG retention. For example, focusing on the day 7 nephrographs, the shift in t_max_ may be an effect of decreased glomerular filtration and the increased in the AUC might be the consequence of uptake by inflammatory infiltrate within the kidney. Additionally nephritic edema, shown by increased kidney wet weight and a significant enlargement of the kidney size on CT images, might have raised the interstitial pressure, which could subsequently narrow the tubules thereby restricting the urine flow and leading to the slower rate of clearance during the excretion phase. While our experiments were not designed to distinguish between these possibilities, these warrant further study. However, the lack of a full mechanistic explanation for our findings may not be necessary before clinical application.

Interestingly, the FDG retention during the late plateau phase was lower for anti-GBM mice on day 7 compared to day 0. While molecular mechanisms were not the main focus of the current work, we did examine expression of the main transporters for FDG in the kidneys. As it has been reported that the use of an SGLT inhibitor increases ^18^F-FDG in urine the decreased expression of SGLTs 1 and 2 is consistent with, but may not be the only cause of this deeper drop [17]. The amplitude of the kidney uptake declined dramatically on days 10, 14, and 21 in reciprocal relationship to sCr and proteinuria, which remained high compared to day 0 levels. A similar lack of correlation between measures of renal function and FDG uptake has been observed in rat models of allogenic transplantation [19]. This further emphasizes the relationship between markers of inflammation and renal retention of FDG.

Many currently available clinical imaging techniques have been applied for the diagnosis and follow-up of lupus nephritis. Ultrasound (US) has been used to evaluate the abnormalities of renal morphology and cortical echogenicity [20]. Other studies have reported the use of diffusion-weighted [21] and T_2_-weighted [22] magnetic resonance imaging (MRI) and duplex doppler sonography [23] for lupus nephritis. Both of these modalities are largely based on morphological changes with some sensitivity in depicting inflammation associated edema directly or indirectly. As with inflammation in other diseases, the inflammatory cells of lupus nephritis are expected to be glucose avid [5]–[7]. Thus we predict FDG-PET would be more sensitive to early changes and therapeutic interventions. Moreover, some patients suffer from claustrophobia and will not undergo MR scanning. Conventional nuclear medicine imaging approaches using ^67^Ga-citrate, ^111^In or ^99m^Tc-labeled leukocytes or IgG have also been used in clinical practice to detect inflammation, but they suffer from major limitations such as prolonged imaging time (up to 48 h or more) and handling of potentially infected blood products. While gallium-67 scintigraphy has shown potential in the assessment of active lupus nephritis based on its affinity for inflammatory lesions [24]–[27], the image quality is less optimal than that of PET and the need for delayed imaging requires at least 2 patient visits delaying diagnosis. In contrast currently nearly all PET scanning is performed on dual modality PET-CT instruments that permit functional and anatomic (changes in renal size and surrounding edema) assessments to be made within a period of about 2 hours. Recently we reported near-infrared (NIR) optical imaging to monitor the renal disease progression in the same anti-GBM nephritis model [28]. The highly overexpressed integrin α_v_β_3_ in nephritis was successfully targeted by the 800CW-RGD dye, showing significant fluorescence intensity from 800CW-RGD dye within nephritic kidneys and persistent retention as long as 14 days post injection. Moreover, the change in the disease course (sCr levels) was paralleled by the change in dye accumulation in the nephritic kidneys. Although the optical fluorescent imaging has unique advantages such as high sensitivity, low cost, and absence of ionizing radiation, one major obstacle to clinical utility is limited tissue light penetration.

Our study is limited by the lack of autoradiography comparing spatially the FDG localization to the inflammatory infiltrate. However, based on its demonstrated uptake in inflammation in other diseases it is reasonable to assume there would be co-localization.

To sum, a non-invasive dual modality imaging technique using FDG-PET-CT has been utilized to serially monitor the status of nephropathy in an experimentally-induced anti-GBM mouse model. In addition to the visual presentation of the changes on PET-CT images, we calculated and evaluated two image derived parameters from the PET-CT data for non-invasive assessment of the disease: a shift in the t_max_ of the renal FDG TAC and a corresponding change in the cumulative FDG retention, i.e. the change in the AUC. Although further studies are needed, these two parameters may be useful in gauging nephritis flares, progression, and regression. They are also practical to measure in humans positioned in a clinical scanner at the time of injection. Given the clinical acceptance of FDG-PET-CT, we believe such a non-invasive analytical tool would facilitate the monitoring and mechanistic understanding of nephritis that arises spontaneously and promote the evaluation of novel therapies. Concerns about radiation are at least mitigated by the clinical need and that the field of view can be restricted to portion of the abdomen containing the kidneys. Finally, our results show kidney FDG uptake correlates with expression of the inflammation and epithelial activation marker VCAM-1. These observations indicate that non-invasive FDG-PET imaging could serve as a harbinger of renal inflammation that sets in prior to the emergence of other clinical and pathological readouts of nephritis.
